# Nickel removal from wastewater using electrocoagulation process with zinc electrodes under various operating conditions: performance investigation, mechanism exploration, and cost analysis

**DOI:** 10.1007/s11356-022-24101-6

**Published:** 2022-11-12

**Authors:** Omar A. Shaker, Safwat M. Safwat, Minerva E. Matta

**Affiliations:** Sanitary & Environmental Engineering Division, Public Works Department, Faculty of Engineering, Cairo University, Giza, 12316 Egypt

**Keywords:** Coagulation, Cost analysis, Electrochemistry, Heavy metals, Kinetics, Wastewater

## Abstract

Economically feasible approaches are needed for wastewater treatment. Electrocoagulation (EC) is an electrochemical treatment method that removes various pollutants from wastewater. It has grown in popularity over conventional treatment methods, especially in industrial wastewater, due to its high performance and the ability to remove toxic compounds. However, it is crucial to reduce the costs associated with EC for widespread implementation. It is also important to decrease nickel (Ni) concentrations in wastewater to prevent potential health and environmental problems. Therefore, this study investigates Ni removal from synthetic and real wastewater using electrocoagulation. Zinc, as a novel electrode, was used as the sacrificial anode. Several operating conditions were assessed, including current density, initial pH, electrolysis time, and spacing between electrodes. The maximum Ni removal efficiency, after 90 min, reached 99.9% at a current density of 10 mA/cm^2^ when the pH was 9.2 and the gap distance was 4 cm. The Ni removal rate reached 94.4% and 94.9% at a 2- and 6-cm spacing, respectively, after 90 min. Anode morphology, kinetic modeling, electrical energy consumption, and cost analysis were also investigated. The type of corrosion was uniform, which is easily predicted compared to pitting corrosion. The comparison between chemical coagulation and electrocoagulation was also reported. Experimental results indicated that the maximum Ni removal rates reached 99.89% after 90 min. The optimum spacing between electrodes was 4 cm, and the optimum current density was 10 mA/cm^2^. Additionally, the kinetic data were best represented through the second-order Lagergren model. The results demonstrated that the electrocoagulation performance was better than that of chemical coagulation for Ni removal. The maximum electrical energy consumption was 23.79 KWh/m^3^ for Ni removal.

## Introduction

Heavy metals have atomic weights ranging from 63 to 200 g/mol. Sources of heavy metals include natural sources, such as geological weathering, and anthropogenic activities, such as those produced by various industries (Ayub et al. 2019; Mariana et al. 2021). Many industries are considered sources of heavy metals, such as metal plating industries, mining processes, tanneries, and paper factories. Wastewater from these industrial practices contains toxic heavy metals, such as nickel (Ni), chromium (Cr), mercury (Hg), cadmium (Cd), arsenic (As), and lead (Pb) (Fu and Wang 2011; Ayub et al. 2020). Heavy metals are hazardous at low concentrations and have carcinogenic effects on human health (Uddin 2017). At high concentrations, nickel is among the most dangerous heavy metals, can have carcinogenic effects on humans and animals, and is reportedly a common cause of allergic contact dermatitis (Cempel and Nikel 2006). Therefore, complete or partial nickel removal from water and wastewater is essential. Decreasing high initial concentrations of heavy metals to acceptable limits remains a technical and economic challenge when using traditional technologies (Sherlala et al. 2018). Among different treatment technologies, chemical methods are considered the most suitable for industrial wastewater treatment due to their ability to handle nonbiodegradable pollutants and provide a reasonable removal rate for toxic materials (Shewa and Dagnew 2020; Zheng et al. 2021; Dolatabadi et al. 2021).

Electrochemical wastewater techniques require substantial investments of capital and have considerable operating costs. However, with the environmental guidelines concerning wastewater discharge, electrochemical methods have proven their worth during the last 20 years (Zhang et al. 2020). One of the promising electrochemical technologies for wastewater treatment is electrocoagulation (EC), which has the benefits of coagulation, flotation, and electrochemistry (Safwat et al. 2019a; Sher et al. 2021). The pollutant removal mechanism in EC depends on electrochemical reactions, chemical reactions, and physical processes, occurring in series or parallel. A simple EC unit consists of two electrodes, submerged in a beaker with the aqueous solution to be treated, externally connected to a power source. The coagulants are generated in situ to neutralize charges and attract and form flocs that float or settle. The generation of metal ions occurs at the sacrificial anode and is accompanied by a hydrogen gas evolution at the cathode, as indicated in the following reactions (Kong et al. 2020):

Anode reactions

1 $${\mathrm{A}}_{(\mathrm{s})}\to {{\mathrm{A}}^{\mathrm{z}+}}_{(\mathrm{aq})}+{\mathrm{ze}}^{-},$$

Cathode reactions

2 $$2{\mathrm{H}}_{2}\mathrm{O}+{2\mathrm{e}}^{-}\to {2{\mathrm{OH}}^{-}}_{(\mathrm{aq})}+{\mathrm{H}}_{2 \left(\mathrm{g}\right)},$$

Reactions within the solution

3 $${{\mathrm{A}}^{\mathrm{z}+}}_{(\mathrm{aq})}+{\mathrm{zH}}_{2}\mathrm{O}\to {\mathrm{A}(\mathrm{OH})}_{\mathrm{z }(\mathrm{s})}+{{\mathrm{zH}}^{+}}_{(\mathrm{aq})},$$

where *A* denotes the metal of the electrode and *z* represents the charge transfer number.

The generation of hydrogen gas assists in floating the flocculated particles. Furthermore, EC is an environmentally friendly process as it requires simple tools and operates easily. The treated wastewater provides acceptable, clear, uncolored, and odorless water, and the sludge produced in EC has the ability to settle and dewater (Gautam and Kumar 2022). The flocs formed in EC resemble chemical flocs, but EC flocs are larger with less solvation water. Moreover, EC can treat wastewater containing heavy metals. Several studies have been conducted using EC to remove Ni from wastewater (Lu et al. 2015; Kim et al. 2020). The uptake of Ni from metal plating wastewater was investigated using EC with iron and aluminum electrodes (Al-Shannag et al. 2015). EC with a monopolar configuration achieved 100% removal for Cr and Ni after a reaction time of 20 min when the current density (CD) was 10 mA/cm^2^ and the pH was 3.0 (Akbal and Camcidotless 2011). In an EC study using a magnesium electrode, the maximum Ni removal rate reached 99% after 30 min when the CD was 0.15 A/dm^2^, and the pH was 7.0 (Vasudevan et al. 2012). A third study revealed that EC with iron electrodes, after 30 min, removed Ni and Cr, and the removal rates reached 96.4% and 96.2%, respectively (Bhagawan et al. 2014). A recent study on EC demonstrated that the maximum Ni removal rates reached 99.75% using an aluminum electrode after 60 min of reaction time with a CD of 20 mA/cm^2^ and a pH of 6.5 (Moersidik et al. 2020).

The performance of EC is significantly affected by the electrode material; thus, it is important to perform a thorough investigation of novel electrodes rather than traditional electrodes (Reilly et al. 2021). Novel electrodes have been recently examined in EC, and researchers have focused on using transition metals because they possess several oxidation states. Previous studies have revealed successful results regarding pollutant removal with transition metal electrodes, such as zinc (Zn). A study on arsenite removal from an aqueous solution using a Zn electrode demonstrated that it eliminated up to 99.89% of the arsenite after 16 min of reaction time at a pH of 6.0 (Ali et al. 2013). A group of researchers investigated boron removal using EC with Zn electrodes, and the removal rate reached 93.2% after 220 min of reaction time at a pH of 7.0 when the CD was 0.2 A/dm^2^ (Vasudevan et al. 2013). Additionally, EC with a Zn anode was applied to remove organic constituents from water in different operating conditions; the removal rate was 84.2% for total phenol and 40.3% for chemical oxygen demand (COD). The filtered olive mill effluent treatment with no added NaCl attained a removal of 72.3% and 20.9% of total phenol and COD, respectively (Fajardo et al. 2015). Furthermore, Pb removal was achieved using a Zn electrode in EC, and the removal rate reached 99.9% when the CD was 1.13 mA/cm^2^ and the pH was 5.68 (Hussin et al. 2017). A recent study conducted in the present laboratory demonstrated that EC with Zn electrodes partially removed urea after 90 min. The removal rate reached 66% with a CD of 21 mA/cm^2^ and a pH of 7.0 (Safwat and Matta 2020).

As a result of limited resources, a reasonable cost method is required to effectively treat wastewater (Safwat et al. 2019c; Mazhar et al. 2021). It is important that EC treatment be affordable for scalability and widespread applicability. Reducing the electricity needed to run the system lowers the overall cost. One approach to achieve this is to assess alternative electrode materials that can effectively remove the desired pollutant while using less current density. In addition, it is important to decrease Ni concentrations in wastewater to prevent potential health and environmental problems. Little information is available regarding electrocoagulation using Zn electrodes for the elimination of heavy metals. As Zn electrodes have exhibited promising results in previous studies, it is essential to investigate EC with Zn electrodes for Ni removal from wastewater as it has a high ability to adsorb, oxidize, and degrade various pollutants, especially micropollutants in domestic and industrial wastewaters. Based on current literature, using EC with Zn electrodes for Ni removal has not been thoroughly studied. Consequently, the primary aims of this research are to examine the efficiency of EC using a zinc electrode to remove Ni and to assess the practicality of the technology from an economic perspective. Specific objectives include (1) system investigation under various operating conditions, including pH, CD, reaction time, and spacing between electrodes, (2) investigating the performance of EC in treating different wastewaters (synthetic and real wastewater) and comparing the performance with chemical coagulation using Zn salts, (3) elucidating the mechanisms of removal through kinetic studies, and (4) illustrating the feasibility of the system through cost analysis. This research will illuminate unknowns concerning the viability of employing transition metals, such as zinc, as an electrode in the EC system for the removal of Ni from wastewater and the costs associated with doing so on a larger scale.

## Materials and methods

### Description of wastewater

Synthetic and real wastewater were experimentally assessed. Synthetic wastewater was prepared using 100 mg/L of nickel nitrate (Ni(NO_3_)_2_) and 1 g/L of sodium chloride (NaCl) (Chem-Lab, Belgium). Real wastewater was obtained from the effluent channel of the primary settling tank of the Abu Rawash wastewater treatment plant in Egypt. The characteristics of the real wastewater for the experiment are described in Table 1. The pH values were adjusted using hydrochloric acid and sodium hydroxide (Advent).

**Table 1:** Wastewater parameters of real wastewater collected from the Abu Rawash treatment plant

Parameter	Value	Unit
pH	6.8	-
Conductivity	970	µs/cm
Chemical oxygen demand	150	mg/L
Total suspended solids	120	mg/L
Total dissolved solids	700	mg/L
Nickel	100	mg/L
Temperature	25	^o^C

### Setup of the electrocoagulation system

**Fig. 1:**
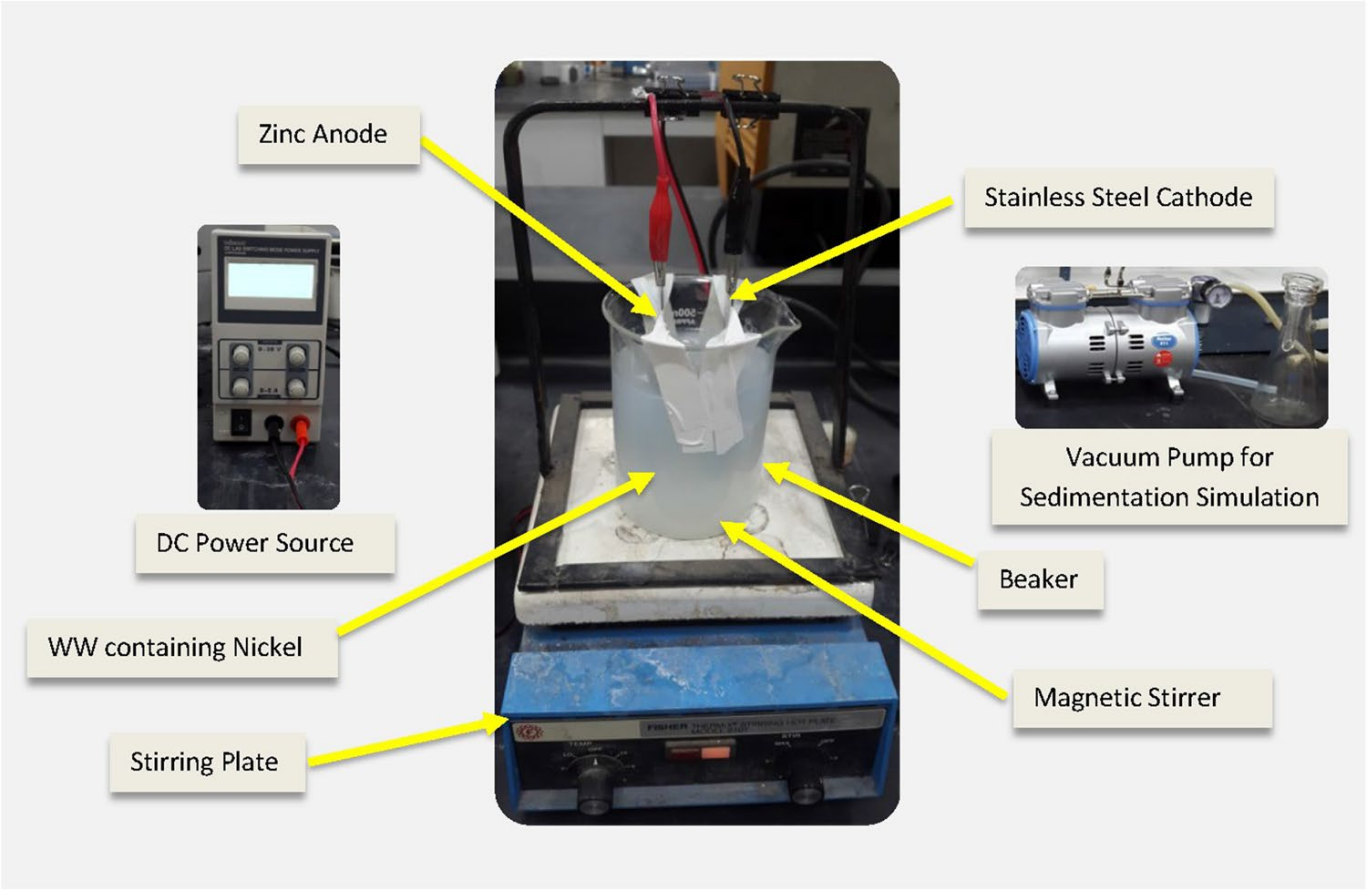
Electrocoagulation (EC) cell setup (WW: wastewater)

The experimental setup consisted of a 500-mL glass beaker in which the electrodes were placed vertically, as presented in Fig. 1. The anode electrodes were 40 × 100 mm Zn plates (> 99%), and the cathode electrodes were stainless steel with the same dimensions. The electrode surface area in the experiments was 80 cm^2^ per plate (double-sided). The inter-electrode distances investigated were 2, 4, and 6 cm. The direct electric current source was connected in a monopolar configuration using a laboratory DC power source (Velleman Energy LABPS3005SM, Belgium). The experiments were conducted at room temperature (25 ± 2 °C). The variable operating conditions were as follows: applied CD (5, 10, and 15 mA/cm^2^), reaction time (1, 5, 10, 15, 30, 45, 60, and 90 min), and initial pH (2.7, 6.8, and 9.2).

The prepared synthetic wastewater in the beaker was stirred at 100 rpm using a magnetic stirrer for each experiment. At the end of every experiment, the treated wastewater was left to settle for 45 min. Then, treated samples were collected and filtered through filter paper using a vacuum pump; sludge was collected for further analysis. Real wastewater samples containing Ni were investigated in EC experiments at the optimum conditions obtained from the synthetic wastewater experiments without altering the pH or adding electrolytes. After each experiment, electrodes were washed with 4% HCl and tap water. Zinc sulfate (ZnSO_4_) served as a coagulant in the chemical coagulation experiments using a jar test apparatus to compare the performance of chemical coagulation with EC. The choice of ZnSO_4_ for chemical coagulation was to simulate the generation of flocs from Zn electrodes in previous EC experiments (Chen et al. 2017; Mamdouh et al. 2021). The following procedures were performed for the chemical coagulation experiments: rapid mixing for 1.5 min at 100 rpm, followed by slow mixing for 20 min at 30 rpm; a settling period of 20 min; and finally, further analysis of the treated samples.

### Analysis

The COD analysis was conducted using a HANNA-COD reactor to measure COD for different water sources. The temperature and pH were measured using an inoLab pH 720 instrument (WTW Series, Germany). Nickel concentrations were measured through ICP (Agilent Technologies, 720 series ICP-OES, USA). Additionally, x-ray fluorescence spectroscopy (XRF; Philips, PW 2404, Germany) was employed to study the residual elements in the sludge after the treatment process. All experiments were conducted in duplicate. Three replicates have been performed for the analyses. The results are reported as averages. The percentage of removal of Ni was calculated in Eq. (4) (Rezgui et al. 2022):

4 $$\%\;R= \frac{{A}_{o}-{A}_{e}}{{A}_{o}} \times 100,$$

where *A*_*e*_ and *A*_*o*_ are the final and initial concentrations of the pollutant, respectively. The Zn electrode morphologies were assessed using scanning electron microscopy (SEM). The following equations (Eqs. (5) and (6)) were used to calculate the sludge production efficiency:

5 $$\eta =\frac{{M}_{exp}}{{M}_{th}}\times 100,$$

6 $${M}_{th}=\frac{M\times I\times t}{n\times f},$$

where *η* indicates the sludge efficiency (%); *M*_*exp*_ denotes the mass of sludge produced experimentally; *M*_*th*_ represents the quantity of dissolved Zn (g); *I* is the current intensity (A); *t* is the reaction time (s); and *M* denotes the specific molecular weight (g/mol). Moreover, Faraday’s constant (*f*) is 96,485 C/mol, and the electron number involved in the reactions is represented by *n*.

The adsorbed Ni ions were calculated using Eq. (7), whereas the adsorbed quantity at equilibrium was estimated using Eq. (8), as follows (Afshin et al. 2021; Safwat et al. 2022):

7 $${q}_{t }=\frac{\left({C}_{o}-{C}_{t}\right)V}{m},$$

8 $${q}_{e }=\frac{\left({C}_{o}-{C}_{e}\right)V}{m},$$

where *q*_*e*_ and *q*_*t*_ represent the quantity of Ni adsorbed (mg/g) at equilibrium and after time *t*, respectively; *V* represents the volume of solution treated (L); *C*_*o*_, *C*_*e*_, and *C*_*t*_ represent the initial concentration of Ni ions (mg/L), the equilibrium of Ni ion concentration (mg/L), and the concentration of Ni ions (mg/L) at time *t*; and *m* represents the mass of the adsorbent (g) (Kumar et al. 2011). Two kinetic models (first- and second-order Lagergren models) were studied (Barjasteh-Askari et al. 2021). The first- and second-order models are generally expressed using Eqs. (9) and (10) (Kamaraj and Vasudevan 2015; Safwat et al. 2019b):

9 $$Log\left(q_e-q_t\right)=Log\left(q_e\right)-{\left(\frac1{2.303}\right)\times}\;k_1t$$

10 $$\frac{1}{{q}_{t}}\times t=\frac{1}{{q}_{e}}\times t+\frac{1}{{k}_{2}{{q}_{e}}^{2}}$$

where *k*_1_ (per min) represents the rate constant of first-order adsorption and *k*_2_ is the rate constant for the second-order kinetic model (g/mg/min) (Kamaraj and Vasudevan 2015).

## Results and discussion

### Investigation of initial pH, current density, and spacing between electrodes

**Fig. 2:**
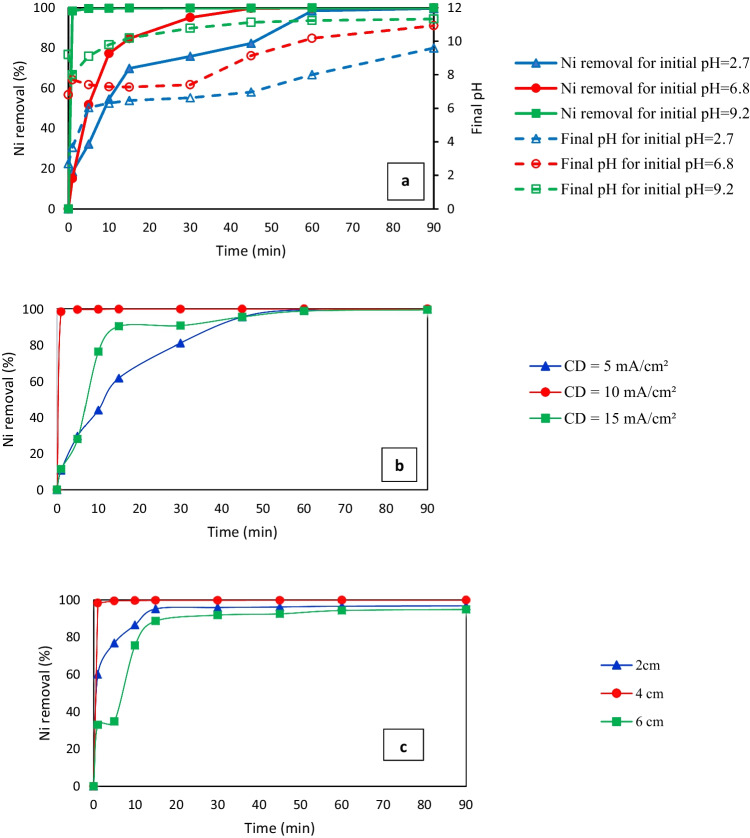
Removal efficiency of nickel versus electrolysis time at different **a** initial pH values (current density (CD) = 10 mA/cm^2^ and spacing = 4 cm), **b** CDs (pH = 9.2 and spacing = 4 cm), and **c** electrode spacings (CD = 10 mA/cm.^2^ and pH = 9.2)

Three main parameters were investigated to optimize the performance of the EC cell. These parameters were various initial pH values, CDs, and spacing between the electrodes. The existence of various species in equilibrium during the operation of EC is related to the initial pH (Garcia-Segura et al. 2017). The effect of the initial pH on EC performance was studied for Ni at a CD of 10 mA/cm^2^, electrode spacing of 4 cm, with NaCl as the electrolyte. The removal rate of Ni for different initial and final pH values is indicated in Fig. 2a. The removal efficiency reached a maximum of 99.9% at the initial pH of 9.2 with an electrolysis time of 90 min. A high Ni removal rate was observed for basic pH values during the first minute, and then the removal rate decreased.

The change in removal rates may be due to oxidation reactions at the anode and stable oxide layers on its surface, causing passivation effects leading to lower removal rates with time (Safwat et al. 2019a). Basic conditions were better than neutral or acidic conditions for Ni removal because basic conditions (pH = 9.2) favor the formation of Zn hydroxide flocs, which act as a coagulant in the pollutant (Ni) adsorption to form complexes. These complexes lead to Ni removal through settling and flotation (Sawyer et al. 2003). These flocs are characterized by their high surface area, which increases the ability to collect pollutants (Abdulrazzaq et al. 2021).

Based on these results, the main removal mechanism is related to adsorption. A high reduction in Ni concentration was obtained after a short reaction time. The removal efficiency of Ni removal reached 99.5% after 5 min; thus, a shorter reaction time can be used, leading to a significant reduction in the energy consumed (Kumar et al. 2004). When the initial pH was 6.8, the removal efficiency reached 99.8% after 45 min, whereas at the initial pH of 2.7 achieved a removal efficiency of 99.4% after 60 min. The increase in pH is due to the generation of H_2_ at the cathode, causing a rise in the OH^−^ concentration in the solution (Hakizimana et al. 2017). This rise enhanced the formation of Zn hydroxide insoluble flocs, assisting the removal of the pollutants. In all cases, the initial pH had little influence on the removal efficiency, provided that the reaction time was more than 60 min.

The generation rate of bubbles and coagulants (floc properties) and the mass transfer of solution are determined through the CD (Lu et al. 2021). Figure 2b presents the removal rates of Ni when using various CDs. Regarding Ni removal, the maximum removal efficiency after 90 min reached 99.9% with a CD of 10 mA/cm^2^. Although the removal rates for other CDs were quite similar after 90 min, they were different. After 1 min, the removal rate of Ni at a CD of 10 mA/cm^2^ almost reached its maximum value compared to other CDs. Increasing the CD can lead to an increase in the Zn dissolution rate, which increases the mixing rate to a level that can negatively affect the formation of flocs. However, decreasing the CD can cause a lower rate of Zn dissolution (Vasudevan et al. 2013). Moreover, energy loss might occur at higher CDs by heating the water (Chen et al. 2018). The maximum Ni removal efficiencies reached 99.4% and 98.7% at CDs of 5 and 15 mA/cm^2^, respectively. Thus, based on the ranges of CD studies, the optimum value was 10 mA/cm^2^. In all cases, the CD within the examined ranges had little influence on the removal efficiency, provided that the reaction time was more than 60 min. Thus, a CD of 10 mA/cm^2^ was used for the subsequent experiments.

The spacing between electrodes has a crucial role in the EC process because the distance between the anode and the cathode affects the electrostatic field (Safwat et al. 2019a). As illustrated in Fig. 2c, three electrode spacings were considered: 2, 4, and 6 cm. The Ni removal rate reached 94.4%, 99.9%, and 94.9% at 2-, 4-, and 6-cm spacing, respectively, after 90 min. For 4-cm spacing, the Ni removal efficiency reached the maximum value during the first min. Then, almost no change happened until the end of the experiments. The heavy metal removal efficiency was low at a smaller electrode spacing (2 cm). This result can be attributed to the metal hydroxides generated in the form of flocs for pollutant removal, which are degraded by collision due to high turbulence from gas generation at the cathode (Hakizimana et al. 2017). Increasing the distance between the electrodes from 2 to 4 cm caused an increase in the removal rate. This increase may be due to the decrease in turbulence. Therefore, the metal hydroxide can agglomerate, leading to the formation of flocs responsible for pollutant removal. Increasing the electrode distance above the value of the optimum electrode distance (increasing the spacing from 4 to 6 cm) caused a considerable decrease in the metal removal rate. This decrease may be caused by the internal resistance of the wastewater, which increases as the distance between electrodes increases (Vasudevan et al. 2013). This decrease resulted in fewer flocs formed than those needed for heavy metal coagulation (Bazrafshan et al. 2015).

### Morphology of electrodes and sludge analysis

**Fig. 3:**
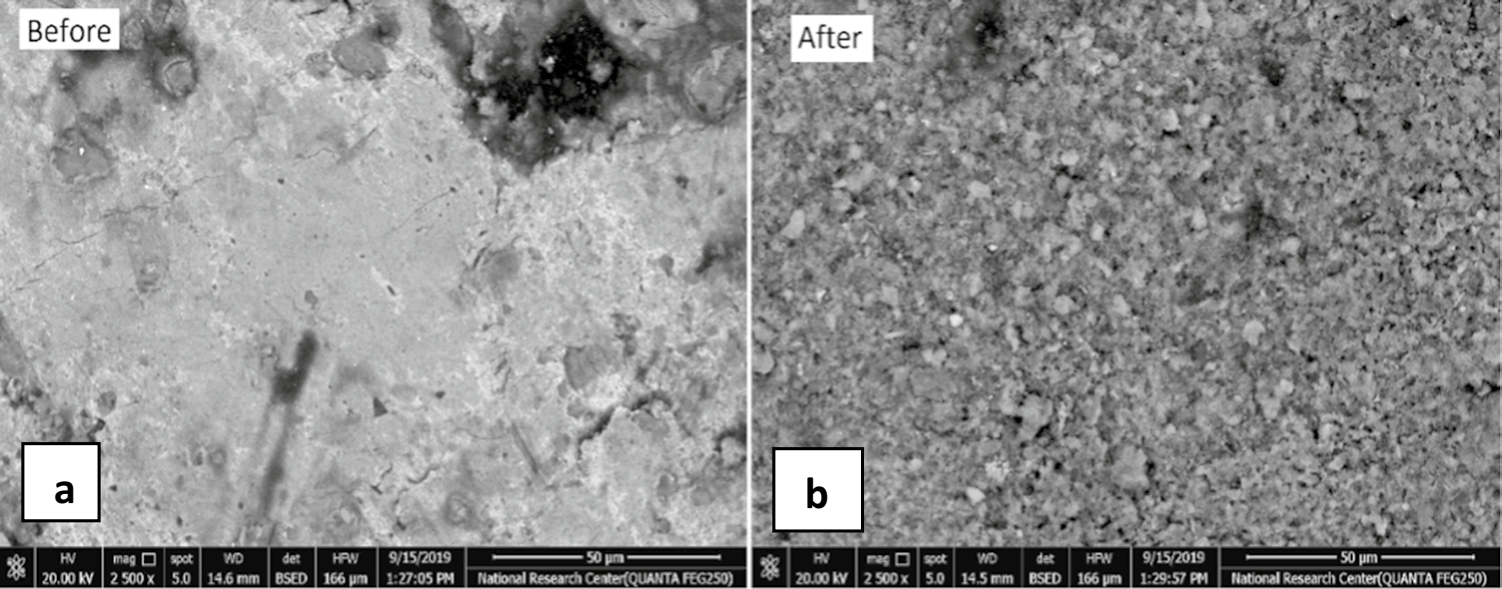
Images of zinc electrodes using scanning electron microscopy: **a** before and **b** after treatment

The anode electrodes were scanned using SEM (Quanta FEG 250, FEI, USA) before and after their use in the EC treatment process at optimum operating conditions (Fig. 3). The anode surface displayed clear corrosion during the removal of Ni. This corrosion proves the occurrence of a loss in the material that ensures the Zn dissolution from the anode to form Zn hydroxides (Safwat et al. 2019a). The type of corrosion was uniform, which is easily predicted compared to pitting corrosion. The uniform corrosion of the electrodes reduces the frequency with which they must be replaced, lowering treatment costs and reducing sludge production (K S and S 2022). Regarding the generated sludge, XRF was used to study the presence of residual elements after the treatment process. The results indicate the existence of Ni and Zn species in the generated sludge. As *M*_*th*_ is 1.46 g/L and *M*_*exp*_ is 1 g/L, *η* was 68%. The variation between the theoretical and experimental values can be attributed to the pH variation during each experiment, affecting the amount of Zn oxide and hydroxides, which are the main components of the generated sludge.

### Removal of nickel from real wastewater

**Fig. 4:**
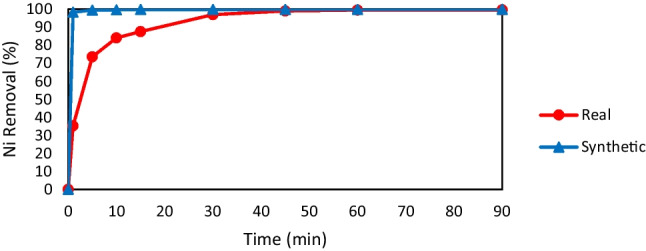
Nickel (Ni) removal efficiency for synthetic and real wastewater vs. reaction time (current density = 10 mA/cm.^2^, pH = 6.8, and spacing = 4 cm)

The behavior of EC with real wastewater is different from that of synthetic wastewater (Yang et al. 2022). Real wastewater containing Ni was treated under the optimum conditions obtained from the previous experiments to illustrate the difference in the system behavior; however, the pH was kept at its actual value of 6.8. The CD value was 10 mA/cm^2^, the spacing was 4 cm, and no salt was added. Table 2 describes the real wastewater characteristics before and after EC treatment. As depicted in Fig. 4, the maximum removal rate of Ni was 99.4%, obtained at 90 min. Most Ni removal was observed during the first 30 min. Afterward, the Ni reduction rate was almost constant. A slightly lower removal efficiency was obtained when using real wastewater. This difference is due to other pollutants that compete for the metal hydroxide generated during the process. The COD values before and after the treatment process showed a 50% removal rate.

**Table 2:** Characteristics of real wastewater after electrocoagulation treatment

Parameter	After	Removal efficiency
pH	11.4	-
Chemical oxygen demand	75 mg/L	50%
Total suspended solids	25 mg/L	79.2%
Total dissolved solids	400 mg/L	42.9%
Nickel	0.6 mg/L	99.4%

### Performance of chemical coagulation

**Fig. 5:**
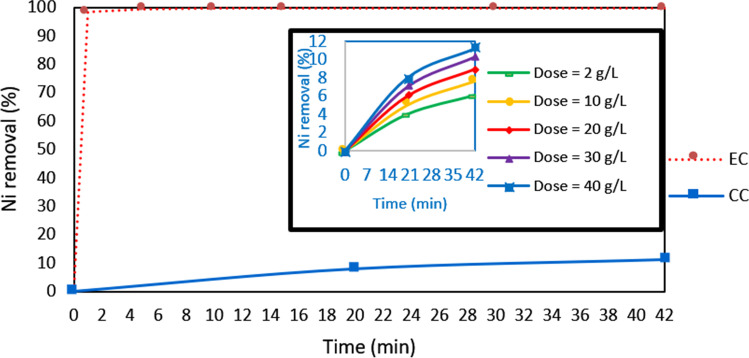
Nickel (Ni) removal rate for electrocoagulation (EC) and chemical coagulation (CC) vs. the reaction time for synthetic wastewater (currently density = 10 mA/cm.^2^, pH = 9.2, and spacing = 4 cm)

Five coagulant doses of ZnSO_4_ (2, 10, 20, 30, and 40 g/L) were used in the jar test apparatus for the chemical coagulation experiments. The maximum efficiency of Ni removal was 11.27% at a coagulant dose of 40 g/L (Fig. 5). The values are less than those corresponding to EC, although the coagulant concentration is considerably high (40 g/L). These results indicate that the EC is superior in performance to chemical coagulation in wastewater treatment. The performance difference between the two systems is related to the mechanism of floc formation. In chemical coagulation, equilibrium conditions govern floc generation, whereas electrochemical reactions and coagulant mass transfer in EC are the main factors governing the formation of flocs (Tegladza et al. 2021a). When different coagulants are generated under different chemical conditions, the coagulation mechanisms and hydroxide floc shape are altered. The use of an electric current in EC has been shown to speed up the breakdown of the zinc electrode and to affect the mass transfer activities of the resulting flocs. The bubbles formed by the hydrogen gas produced during the hydrolysis reaction at the cathode aid in the precipitation of flocs and in the treatment process through the flotation phenomena (Tegladza et al. 2021b).

### Mechanism and kinetic modeling

**Fig. 6:**
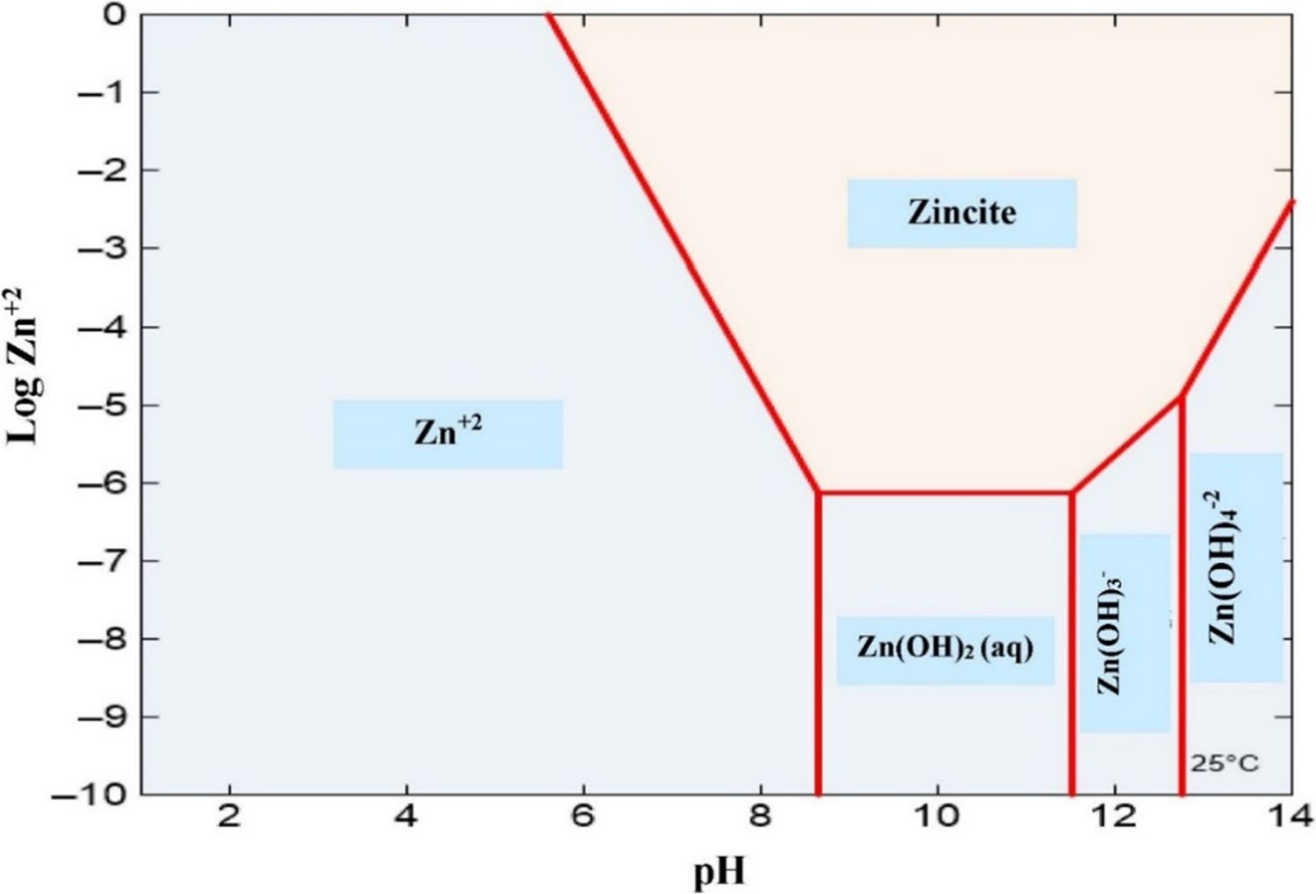
Predominance zone diagram for zinc species in an aqueous solution

The predominance zone diagram of Zn(II) is presented in Fig. 6. This figure illustrates that zincite (ZnO) coagulates when pH is greater than 6.0. Afterward, according to the pH range, the predominant precipitation species exists in equilibrium with different soluble monomers. Thus, ZnO is in equilibrium with Zn^+2^ for pH values up to 8.5, with Zn(OH)_2_ for values from 8.5 to 11.5, with Zn(OH)_3_^−^ for 11.5 − 12.8, and with Zn(OH)_4_^−^ for 12.8 − 14. The pH increases during the EC experiments regardless of the initial pH. The availability of sufficient coagulant in the solution and the minimum solubility of Zn oxide in this pH range may be why the pollutant removal efficacy increased.

**Fig. 7:**
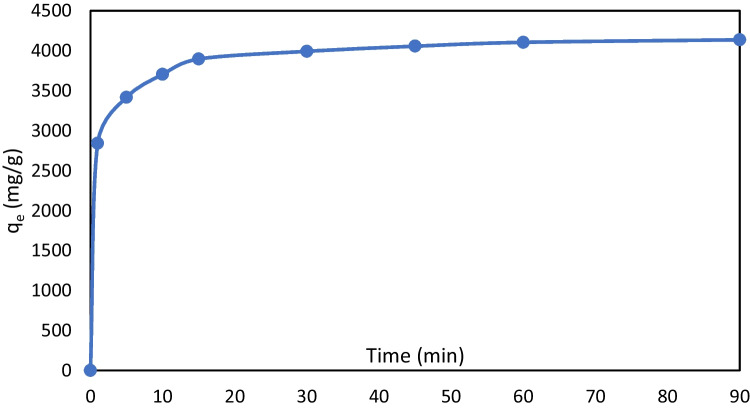
Effect over time on adsorbed nickel and *q*_*e*_ at 100 mg/L (current density = 10 mA/cm.^2^, pH = 9.2, and spacing = 4 cm)

Various forms of Ni in the solution can be removed by several mechanisms during the treatment time, depending on the pH, including adsorption, electrostatic attraction, precipitation, and sweep flocculation. Kinetic modeling was used to report the reaction rates for various CDs. The equilibrium time was 60 min for all examined CDs (Fig. 7). After 60 min, adsorbed Ni increased from 4105 to 4137 mg/g, when the CD changed from 5 to 15 mA/cm^2^. A single, smooth, continuous curve in the plot suggests the possibility of covering the monolayer on the adsorbent surface. The results from the first and second models are presented in Table 3, demonstrating that the experimental data were best expressed with a second-order Lagergren model (high coefficient of determination). Moreover, *q*_*e*_ (calc) and *k*_2_ were determined from the slope and intercept of the graph of *t*/*q*_*t*_ versus *t*. Further, *q*_*e*_ (calc) is consistent with the experimental values of *q*_*e*_ (exp) in all studied CDs. Therefore, the second-order model effectively explains Ni adsorption in the produced flocs.

**Table 3:** Experimental and calculated *q*_*e*_ values at current densities in first- and second-order kinetic models of nickel at an initial concentration of 100 mg/L

Pollutant	Current density (mA/cm^2^)		First-order model			Second-order model		
		*q*_*e*_ (mg/g) (exp)	*q*_*e*_ (mg/g) (calc)	*k*_1_ (per min)	*R*^2^	*q*_*e*_ (mg/g) (calc)	*k*_2_ (g/mg/min)	*R*^2^
Nickel	5	64	375	− 0.042	0.68	69	− 0.0021	0.92
	10	32	889	− 0.074	0.48	35	− 0.0034	0.91
	15	21	169	− 0.047	0.65	23	− 0.0054	0.92

This study showed a high Ni removal efficiency from the aqueous solution. Similar removal efficiencies for other heavy metals were obtained in previous studies. The zinc removal efficiency of the aqueous solution reached 99.9% when the hybrid process with activated carbon fiber and micellar enhanced ultrafiltration hybrid process (Channarong et al. 2010). When the Fered-Fenton and chemical precipitation process were used, the Ni removal efficiency was 99.9% (Shih et al. 2013). In addition, industrial coal fly ash-nano zerovalent iron was able to remove 99.9% of cadmium from the aqueous solution (Ma et al. 2018). Furthermore, ferric ions were removed using a new sulfidogenic acid mine drainage treatment system, and the removal efficiency reached 99.9% (Sun et al. 2020). Additionally, 99.9% of lead was removed during leaching experiments in the NaOH-S system (Zhang et al. 2021). Similarly, electrocoagulation was able to remove 99.9% of iron from wastewater (Beiramzadeh et al. 2022). Although all these studies showed a 99.9% removal efficiency for the target heavy metal, the removal efficiency of the treatment process should not be used as the sole factor to assess the success of the process. It is important to consider the residual concentration of the pollutant after the treatment process. Thus, the findings of this study can fill some of the literature gaps, but further research will be needed to examine other parameters, such as the effect of the initial concentration on the performance of the system. Furthermore, additional investigation is needed with respect to the effect of competition with other pollutants during the removal process.

### Electrical energy consumption and cost analysis

**Fig. 8:**
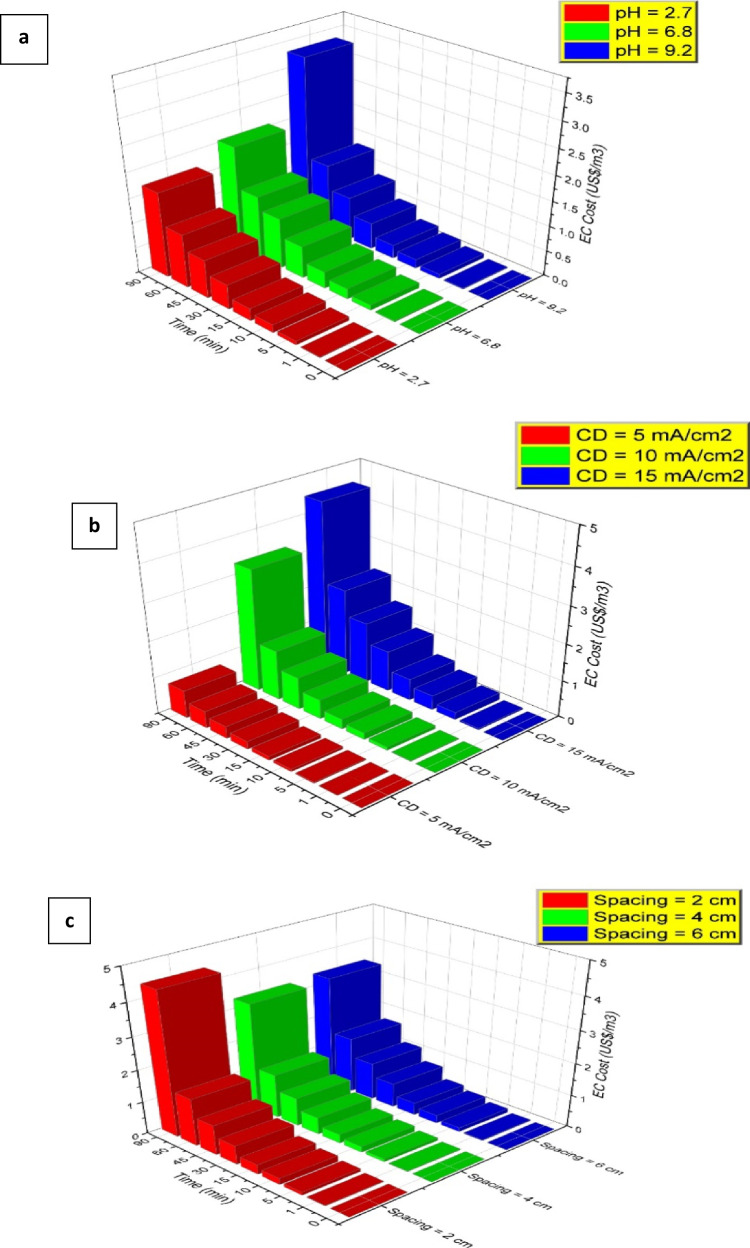
Cost variation for synthetic wastewater containing **a** nickel (Ni) vs. initial pH (current density (CD) = 10 mA/cm^2^ and spacing = 4 cm), **b** Ni vs. different CDs (pH = 9.2 and spacing = 4 cm), and **c** Ni vs. various spacings (CD = 10 mA/cm.^2^ and pH = 9.2)

The optimization process for any technology must consider the operational cost as it is an essential factor that determines the feasibility of the process at the industrial level (Garcia-Segura et al. 2017). For EC systems, it is important to evaluate the performance according to the CD value and consider the EC time because a low CD can achieve good removal rates but requires a long electrolysis time (Chen et al. 2018). The operational costs of an EC reactor (EC cost) for the treated effluent can be obtained by considering two main terms: the energy consumption value and amount of Zn electrode that is scarified. The material cost considers the theoretical maximum possible mass of Zn produced from the anode, obtained from Faraday’s law, per cubic meter of the treated effluent, and the cost of the electrode material (*EMP*) as USD per gram of Zn. Electrical energy consumption is the highest operating cost in the EC process. Thus, optimization to reduce the cost and environmental effects is critical (Fajardo et al. 2015). The electrical operational cost (*EOC*) and the material cost (*MC*) can be calculated by using the following equations (Eqs. (11) and (12)):

11 $$EOC= \frac{V \times i \times t}{{V}_{eff}} \times EEP,$$

12 $$MC= \frac{M \times i \times t}{{V}_{eff} \times n \times f} \times EMP,$$

where *EOC* represents the operating cost due to consumed electricity (USD/m^3^); *V* indicates the potential difference (V); *t* is the time (h); *V*_*eff*_ denotes the total volume (m^3^); *EEP* represents the cost of the electrical energy (USD/kWh); *MC* denotes the material cost (USD/m^3^); *EMP* indicates the electrode material price (USD/g); and *M* denotes the relative molar mass of the Zn electrode (g/mol) (Espinoza-Quiñones et al. 2009). Figure 8 reveals that the EC cost is calculated at different reaction times for each parameter, confirming the achievement of the optimum conditions with the lowest cost. Regarding time, 60 min achieves the highest removal with the lowest cost. The electrical energy consumption depends on the electrical current and reaction time, so the higher the CD and reaction time, the higher the value of *EEC*. The results revealed that the least EC cost was around 0.7 USD/m^3^ when the CD was 5 mA/cm^2^ with a spacing of 4 cm and an initial pH of 9.2. The Ni removal efficiency associated with this cost after 90 min was 99.7%. Based on these results, it is more economically feasible to use a CD of 5 mA/cm^2^ instead of 10 mA/cm^2^ because the difference in the removal efficiency is negligible. Moreover, the reaction time can be reduced to only 60 min instead of 90 min. In this case, the removal efficiency of Ni is 99.4%, and the associated EC cost is 0.5 USD/m^3^.

## Conclusion

This study investigated the performance, mechanism, and cost analysis of using Zn electrodes in EC to remove Ni from wastewater. The results revealed high removal rates during the first 60 min. The removal efficiency increased with increasing initial pH, whereas the removal efficiency decreased when changing the gap distance between electrodes (2 and 6 cm). The optimum conditions within the study parameters were a CD of 10 mA/cm^2^, an initial pH of 9.2, and a 4-cm gap between electrodes. Because the electrodes corrode evenly, they may go longer between replacements, minimizing both treatment costs and sludge generation. Furthermore, EC treated real wastewater containing Ni despite the competition with other pollutants. The Ni removal rate reached 99.9% for synesthetic wastewater and 99.4% for real wastewater. When operating the EC using real wastewater, the COD levels before and after treatment reduced by 50%. Kinetic studies revealed that the experimental data best fit the second-order model with a coefficient of determination of more than 0.9. The morphology of the Zn electrodes after the treatment process through SEM images indicated uniform corrosion from the EC process. When comparing the performance with chemical coagulation, EC was superior. The results also indicate that electric energy consumption increases with time. However, the economically feasible CD that can be used was found to be 5 mA/cm^2^. Additionally, the 90-min operation can be reduced to 60 min. The EC cost was $0.5/m^3^, and the efficiency of Ni removal was 99.4%. This study found that Zn electrodes are successful in removing nickel from wastewater through the simple configuration of the EC process. It should be noted that, despite the high Ni removal efficiency obtained, it is essential to take into account the residual concentration in the effluent after the treatment process. Given the evidence of the concepts presented in this study as well as the results, the knowledge gap highlights the need for further research to advance investigation in this field. The practical aspects of EC and other parameters must be studied to ascertain the influence of their implementation. Further investigation of the effects of various concentrations of Ni on the performance of the system is warranted. In addition, the effect of various configurations and the existence of other heavy metals in the system requires in-depth examination. Moreover, sludge management is an important aspect during the treatment process. The availability of these data can improve the performance of the system in full-scale applications.

## Data Availability

All data generated or analyzed during this study are included in this published article.
